# Risk factors for non-fusion segment disease after anterior cervical spondylosis surgery: a retrospective study with long-term follow-up of 171 patients

**DOI:** 10.1186/s13018-018-0717-1

**Published:** 2018-02-02

**Authors:** Ziqiang Wang, Liangliang Zhou, Bin Lin, Keran Song, Qinghe Niu, Dongfeng Ren, Jiaguang Tang

**Affiliations:** 1 Jinzhou Medical University, Jinzhou, 121001 People’s Republic of China; 2 Department of Orthopedics, The First Affiliated Hospital of the General Hospital of PLA, Beijing, 100048 People’s Republic of China

**Keywords:** Non-fusion segment disease, Anterior cervical arthrodesis, Cervical spondylosis, Adjacent segment disease

## Abstract

**Background:**

The purpose of this study was to investigate the incidence and causes of non-fusion segment disease (NFSD), both adjacent and non-adjacent to a fused segment, after anterior cervical arthrodesis.

**Methods:**

This is a single-center study. Between January 1998 and January 2011, two surgeons’ 171 patients who had an anterior cervical decompression and fusion were followed clinically for more than 5 years. The correlation between the incidence of symptomatic non-fusion segment disease and the following clinical parameters (age at operation, fusion levels,) and radiological parameters (number of patients who had a plate, anterior cervical decompression and fusion (ACDF) or corpectomies, preoperative and postoperative cervical spine alignment, Pavlov’s ratio at the C5 level, and preoperative existence of a non-fusion segment degeneration on magnetic resonance imaging) was evaluated.

**Results:**

Of the 171 patients reviewed, 16 patients had non-fusion segment disease (9.36%), of which 12 had adjacent segment disease and 4 had non-adjacent segment disease. Postoperative cervical lordosis in the non-fusion segment disease group was significantly smaller than that of the disease-free group (*P* < 0.001). Fusion levels in the NFSD group were 1.69 whereas 2.26 in disease-free group (*P* = 0.005). The incidences of disc degeneration in unfused segments was more severe in the NFSD group than in the disease-free group (*P* = 0.004). The results of binary logistic regression showed that the major factor affecting NFSD is postoperative cervical lordosis (*P* = 0.000) followed by disc degeneration (*P* = 0.024). The other parameters did not show a statistically significant difference.

**Conclusions:**

The incidence of symptomatic non-fusion segment disease after anterior cervical arthrodesis has multifactorial causes. Postoperative cervical lordosis and disc degeneration in non-fusion segments were major factors in the incidence of NFSD.

## Background

For more than 50 years, since its introduction by Smith and Robinson [1, 2], anterior cervical decompression and fusion has been an effective treatment for myelopathy and radiculopathy induced by a degenerated and herniated intervertebral disc. Many follow-up studies have demonstrated its excellent neurological outcomes. However, radiographic studies have shown that the disc adjacent to the fused spinal segment degenerated in 50 to 90% of patients on long term follow-up [3–5]. The occurrence of these degenerative changes may lead to new symptoms known as adjacent segment disease (ASD), the incidence of which ranges from 5.1 to 21% according to previous reports [6–11]. However, the cause of the ASD, be it increased intradiscal stress in the adjacent segment, the natural process of degeneration, or other factors, remains unknown. The incidence of new symptoms at non-adjacent levels is also unknown. The purpose of the present work is to investigate the incidence and causes of non-fusion segment disease (NFSD), both adjacent and non-adjacent to a fused segment, after anterior cervical arthrodesis.

## Methods

Between February 1998 and February 2011, two surgeons’171 patients who had an anterior cervical decompression and fusion for intervertebral disc herniation and cervical spondylosis were followed clinically for more than 5 years. Of these, 97 were men and 74 were women and the average age at operation was 51.9 ± 9.28 (range, 31 to 72 years). The average length of follow-up was 8.70 ± 3.16 years (range 5 to 13 years).

## Surgical technique

Of the 171 patients, 31 had one, 80 had two, 53 had three, and 7 had four level fusions. The anterior cervical decompression and fusion was performed according to the Smith-Robinson technique [1, 2]. Patients with one level decompression and fusion received one intervertebral implant. Patients with two to four levels decompression and fusion received one intervertebral implant per level plus an anterior cervical plate, multilevel corpectomies plus an anterior cervical plate were also included in the cohort. The average duration of collar treatment was 8 weeks, after which patients returned to moderate activities and were followed radiographically.

## Radiographic and clinical evaluation

Follow-up information collected at clinic visits included postoperative symptoms, neurological examination, and radiographs. Patients with new symptoms received MRI examination. Diagnosis of symptomatic NFSD was based on the presence of both new radiculopathy or myelopathy symptoms referable to the levels, and a compressive lesion at the same levels on MRI. The outcome of NFSD was evaluated according to the criteria of Hilibrand et al. [9]. Clinical parameters used to evaluate the incidence of NFSD were age at operation and fusion level(s). Radiological parameters used were number of patients who had a plate, ACDF or corpectomies, preoperative and postoperative cervical spine alignment, Pavlov’s ratio at the C5 level, and the presence of non-fusion segment disc degeneration on preoperative MRI. Cervical spine alignment was measured as the angle between C2 and C7 on lateral, and standing radiograph. Postoperative cervical spine alignment was measured on the final follow-up radiograph. To facilitate inter-patient comparison of the severity of non-fusion segment disc degeneration, MRI’s were evaluated by a 5-grade classification system. Grade 0: no degeneration, grade 1: degeneration without dural compression, grade 2: degeneration with subdural space compression, grade 3: degeneration with subdural space absent, and grade 4: degeneration with spinal cord compression.

## Statistical analysis

All data were collected. Statistical analysis was performed using SPSS 17.0 (SPSS Inc., Chicago, IL, USA). The independent two-sample *t* test was used to compare the preoperative, postoperative, and final follow-up clinical and radiographic data. Counting data using chi-square test. Binary logistic regression was used to make definitive conclusions about independent predictors of NFSD. All results were reported as means ± standard deviation (SD). Significance was defined as *P* < 0.05.

## Results

**Fig. 1:**
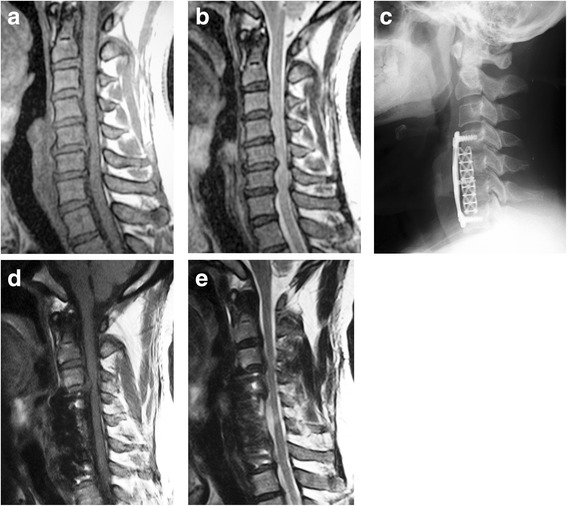
47-year-old man with multilevel cervical intervertebral disc herniation. **a**, **b** Preoperative T1-weighted and T2-weighted MRI shows an indentation of dura mater at C3-C4, C4-C5, C5-C6 and C6-C7 levels. **c** Radiograph after operation shows 3-level fusion from C4-C7. **d**, **e** MRI at 6 years after operation indicates complete decompression at C4-C5, C5-C6, and C6-C7 levels, but C3-C4 level shows significant spinal cord compression

**Fig. 2:**
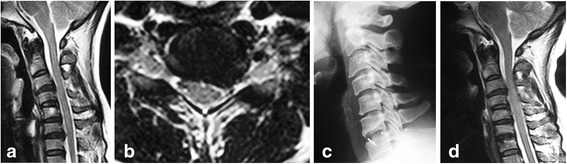
39-year-old female with C6-C7 cervical intervertebral disc herniation. **a**, **b** Preoperative T2-weighted MRI shows left nerve root compression at C6-C7 level and disc degeneration at C3-C4 level. **c** Radiograph after operation shows C6-C7 fusion. **d** MRI at 1 year after operation indicates complete decompression at C6-C7, but severe spinal cord compression can be seen at C3-C4 level

NFSD occurred in 16 patients (9.36%), 9 were male and 7 were female, there is no difference between male and female using chi-square test (*P* = 0.968). Of these, 12 had adjacent segment disease (typical cases are shown in Fig. 1) and 4 had non-adjacent segment disease (typical cases are shown in Fig. 2).The average length of time from first operation to the onset of symptomatic NFSD was 5.00 ± 2.83 years (range 1 to 13 years). The average age at first operation was 51.06 ± 7.15 years (range 38 to 64 years) in NFSD group and 51.06 ± 9.92 years (range 31 to 72 years) in disease-free group. There was no difference between the two groups (*P* = 0.844).

Preoperative alignment in the NFSD group was 7.75°± 3.44 and in the disease-free group was 9.32°± 3.79, which was not statistically different when evaluated by *t* test. However, postoperative alignment was significantly different (*P* < 0.001) between the two groups with the NFSD group measuring 8.38°± 4.57 versus 17.30°± 4.43 in the disease-free group. Pavlov’s ratio did not show significant difference between groups, with the NFSD group measuring 0.84 ± 0.08 versus 0.83 ± 0.09 in the non-NFSD group (*P* = 0.597). The number of levels fused showed a significant difference between the two groups (NFSD group = 1.69 ± 0.79 levels and disease-free group = 2.26 ± 0.77 levels, *P* = 0.005). The scores of disc degeneration in preoperative MRI at the non-fusion segments were significantly higher in NFSD group (NFSD group = 2.25 ± 1.13 and disease-free group = 1.52 ± 0.94, *P* = 0.004). Table 1 shows the difference between the NFSD group and disease-free group using *t* test. Multilevel corpectomies total 17 cases, 3 had NFSD, ACDF 154 cases, 13 had NFSD, the chi-square test of continuity correction showed no significant difference between the two groups (*P* = 0.425). The number of patients who had a plate was 87, 8 had NFSD, there was no statistical difference in NFSD between patients who had a plate or not (*P* = 0.941). Table 2 shows the difference between the NFSD group and disease-free group using chi-square test. The results of binary logistic regression showed that the major factor affecting NFSD is postoperative cervical lordosis (*P* = 0.000) followed by disc degeneration (*P* = 0.024). The other parameters did not show a statistically significant difference (Table 3).

**Table 1:** The difference between the NFSD group and disease-free group using t test

	NFSD group(*n* = 16)	Disease-free group(*n* = 155)	*P* value
Age at operation (years)	51.06 ± 7.15	50.56 ± 9.92	0.844
Preoperative alignment (degrees)	7.75 ± 3.44	9.32 ± 3.79	0.113
Postoperative alignment(degrees)*	8.38 ± 4.57	17.30 ± 4.43	0.000
Pavlov’s ratio (C5)	0.84 ± 0.08	0.83 ± 0.09	0.597
Number of levels fused*	1.69 ± 0.79	2.26 ± 0.77	0.005
Scores of disc degeneration in non-fusion segments*	2.25 ± 1.13	1.52 ± 0.94	0.004

Note: * for the difference was statistically significant (*P* < 0.05)

**Table 2:** The difference between the NFSD group and disease-free group using chi-square test

	NFSD group (n = 16)	disease-free group (*n* = 145)	*P*
Sex			
Male	9	88	0.968
Female	7	67	
Plate			
Yes	8	79	0.941
No	8	76	
surgery			
Corpectomies	3	14	0.425
ACDF	13	141

**Table 3:** Binary logistic regression model for NFSD

	Exp (B) (95% CI of Exp (B))	*P*
Age	1.039(.947–1.140)	0.419
Sex	0.916(.142–5.895)	0.926
Pavlov’s ratio	0.010(.000–49.518)	0.288
Scores of disc degeneration*	0.360(.149–.873)	0.024
surgery	1.570(.094–26.111)	0.753
Number of levels fused	2.321(.779–6.915)	0.130
plate	2.233(.392–12.722)	0.366
Postoperative alignment*	1.592(1.262–2.008)	0.000
Preoperative alignment	0.966(.767–1.215)	0.767

Note: * for the difference was statistically significant (*P* < 0.05)

## Discussion

In this retrospective study postoperative cervical spine alignment, severity of non-fusion segment degeneration, and number of the levels fused are all factors contributing to new symptomatic NFSD. Therefore, NFSD has a multivariate etiology.

There are few articles in the literature which focus on the importance of sagittal alignment and its relation to the development of ASD in the cervical spine. Katsuura et al. [12] noted that after anterior cervical fusion 43% of patients with ASD had malalignment of the cervical spine at the time of diagnosis. Degenerative changes in adjacent intervertebral levels were observed in 77% of segments fused in kyphosis. Kumar et al. [13] studied the relationship between lumbar sagittal imbalance and the development of ASD. They found that patients with sagittal imbalance or/and vertical sacral inclination had a 50% incidence of ASD, much higher than patients with normal sagittal alignment and vertical sacral inclination. Biomechanical studies have also demonstrated the impact of sagittal alignment on ASD [14]. Our study also revealed that the degree of cervical lordosis in the NFSD group was significantly smaller than that of the disease-free group, supporting the theory that cervical spine alignment after fusion impacts NFSD and that maintenance of normal cervical spine curvature might decrease the incidence of NFSD. Cervical alignment is a factor that needs to be considered during the operation. The methods we used to correct the alignment include the following: 1, surgical position (hyperextension position); 2, the height of the implant used should be slightly higher than the actual vertebral space by 1 mm; 3, neck pain may cause the cervical alignment to straight, after decompression, the patient’s pain was relieved and the alignment was corrected.

In our study, preoperative MRI revealed that disc degeneration in unfused segments was more severe in the NFSD group than in the disease-free group. This result is somehow in consistent with Hilibrand’s studies [9]. Follow-up of 374 patients for up to 21 years, Hilibrand et al. found a correlation between poor initial radiological grades at adjacent levels and subsequent development of ASD. In a study by Ishihara and colleagues [15], the incidence of symptomatic ASD after ACDF was higher when preoperative myelography or MRI revealed asymptomatic disc degeneration at that level. Their findings suggest that this disease is a result of a continuing degenerative process rather than a late complication of fusion. This theory is further supported by the finding that the disease is far less common among patients who had undergone cervical fusion for trauma than for degenerative disease [16]. As a further support of the effect of the natural degenerative process on non-fusion segment disease, our study showed symptomatic non-adjacent segment disease in four patients whose preoperative MRI showed degenerative changes in these non-adjacent segments. This result was similar to that of RAO et al. [4], who studied a large series of patients with anterior cervical fusion and found that preoperative cervical degenerative changes will affect the occurrence of postoperative degeneration. Jack et al. [17] also found that the existence of cervical degenerative changes in patients with a high rate of repair after surgery. SONG et al. [18] conducted a follow-up study of 87 patients undergoing anterior cervical decompression and fusion, suggesting that the occurrence of adjacent segmental disease was more likely to be the natural degeneration of the disc itself. The results of lumbar fusion studies have similar results. Natarajan [19] studied the effects of lumbar degeneration on adjacent segmental motion through a finite element analysis model, suggesting that intervertebral disc degeneration was a risk factor of adjacent segmental lesions after lumbar fusion. The concept of “motion preservation” technology has led to the development of cervical total disc arthroplasty (TDA). However, in a prospective, randomized, FDA investigational device exemption (IDE) trial, which was the 2010 Spine Journal “Outstanding Paper,” Jawahar et al. [20] did found no significant difference between TDA and ACDF for ASD, but reported that the ASD is significantly higher in patients with concurrent degenerative disc disease in the lumbar spine. Chang et al. [21] reached the opposite conclusion, in the analysis of the incidence of ASD after ACDF and TDA, the latter (3.1%, 0–7.1%) was lower than the previous (6.0%, 1.0–11.9%), but said further studies were needed.

In clinical practice, the authors have noted that the majority of patients treated for cervical spondylotic radiculopathy or myelopathy have degenerative changes in more than one segment. Considering that the majority of patients improve after decompression of the symptomatic segments, most surgeons would not sacrifice asymptomatic “innocent” segments. However, some of these patients will indeed develop NFSD. Therefore, it is difficult to decide if those “innocent” segments should be fused. Increasing fusion levels can indeed avoid ASD. This study has shown that in the disease-free group, 2.26 segments were fused on average whereas 1.69 segments were fused in the NFSD group.

Some studies have suggested that increasing fusion levels is thought to increase the burden of adjacent segments and is more likely to cause ASD. Chung et al. [22] analyzed the incidence of ASD in 177 patients and found that patients receiving multi-segmental fusion (32.1%) had a higher incidence of ASD than single segment (13.2%), suggesting that the combined biomechanics may cause changes in adjacent segments. Bydon et al. [23] considered that iatrogenic introduction leads to stress and instability in adjacent segments, which is associated with the occurrence of ASD.

But there are also similar research results finding with us. Hilibrand et al. [9] reported that the incidence of ASD decreased after multilevel fusions. Rao [4] found that the fusion segment had no effect on the degeneration of adjacent segments. Louie et al. [5] also reported the incidence of ASD in patients with single-segment or multi-segment cervical ACDF surgery, which suggests that multi-segment cervical ACDF did not increase the risk of ASD. In addition, Lee et al. [24] found that the risk of ASD in one or two segments of cervical fusion was 1.8 times higher than the risk of involving three or more segments fusion. Lee suggested that in most cases, arthrodesis should involve all necessary levels but be limited to as few levels as possible. These suggest that ASD is not only solely due to the arthrodesis but also involves the natural history of spondylotic disease [25]. It is probably unfair to suggest that there may be an indication to fuse more levels based on the results of *t* test, which in logistic regression, fuse level is not the major factor affecting NFSD. However, a multilevel fusion could provide some degree of protection. This is a further verification of natural degenerative changes playing an important role in NFSD.

## Limitation

Our study has some limitations. This study was only a retrospective study with a small sample size to explore the risk factors for non-fusion segment disease after previous cervical spine fusions. Furthermore, cervical total disc replacement was not included in this study. Cervical degeneration is a result of multiple factors that require further study of more factors such as psychology, job, and diet.

## Conclusions

In conclusion, our study has demonstrated that NFSD has multifactorial causes. Cervical lordosis post-op, disc degeneration in asymptomatic segments were all factors in the incidence NFSD. In order to prevent NFSD, surgeons must consider all of these factors when performing anterior cervical fusion.

## Abbreviations

ACDF: Anterior cervical discectomy and fusion
ASD: Adjacent segment disease
MRI: Magnetic resonance imaging
NFSD: Non-fusion segment disease
TDA: Total disc arthroplasty
